# Useful Effects of Melatonin in Peripheral Nerve Injury and Development of the Nervous System

**DOI:** 10.1055/s-0036-1597838

**Published:** 2017-02-16

**Authors:** Yigit Uyanikgil, Turker Cavusoglu, Kubilay Dogan Kılıc, Gurkan Yigitturk, Servet Celik, Richard Shane Tubbs, Mehmet Turgut

**Affiliations:** 1 Department of Histology and Embryology, Faculty of Medicine, Ege University, İzmir, Turkey; 2 Cord Blood, Cell-Tissue Research and Application Center, Ege University, İzmir, Turkey; 3 Department of Anatomy, Faculty of Medicine, Ege University, İzmir, Turkey; 4 Seattle Science Foundation, Seattle, Washington, United States; 5 Department of Neurosurgery, Adnan Menderes University School of Medicine, Aydın, Turkey

**Keywords:** melatonin, peripheral nerve, nerve injury, pineal gland

## Abstract

This review summarizes the role of melatonin (MLT) in defense against toxic-free radicals and its novel effects in the development of the nervous system, and the effect of endogenously produced and exogenously administered MLT in reducing the degree of tissue and nerve injuries. MLT was recently reported to be an effective free radical scavenger and antioxidant. Since endogenous MLT levels fall significantly in senility, these findings imply that the loss of this antioxidant could contribute to the incidence or severity of some age-related neurodegenerative diseases. Considering the high efficacy of MLT in overcoming much of the injury not only to the peripheral nerve but also to other organs, clinical trials for this purpose should be seriously considered.

## Introduction

Melatonin (MLT), N-acetyl-5-methoxytryptamine, is a secretory product synthesized nocturnally by the pineal gland. Its useful effects include modulation of pituitary hormones, stimulation of the immune system, and photoperiod adaptation. 1 2 Fetal brain tissue does not secrete it, but the fetus obtains sufficient for its needs via placental transfer from the maternal system. 3

Peripheral nerve injury (PNI) can result in demyelination, axonal degeneration, or both. Peripheral nerve injuries can cause motor and sensory function losses. Depending on the severity and degree of nerve injury, function recovers with remyelination and with axonal regeneration and reinnervation of the sensory receptors or motor end plates. Seddon classified nerve injury degree according to severity. 4

The central nervous system (CNS), composed of the brain and spinal cord, integrates received information and coordinates the activities of all parts of the body. Some classifications of the CNS additionally include the retina and cranial nerves. In conjunction with the peripheral nervous system (PNS), it is important in controlling behaviors. 5

## Anatomy, Physiology, and Histology of the Pineal Gland and Melatonin

**Fig. 1:**
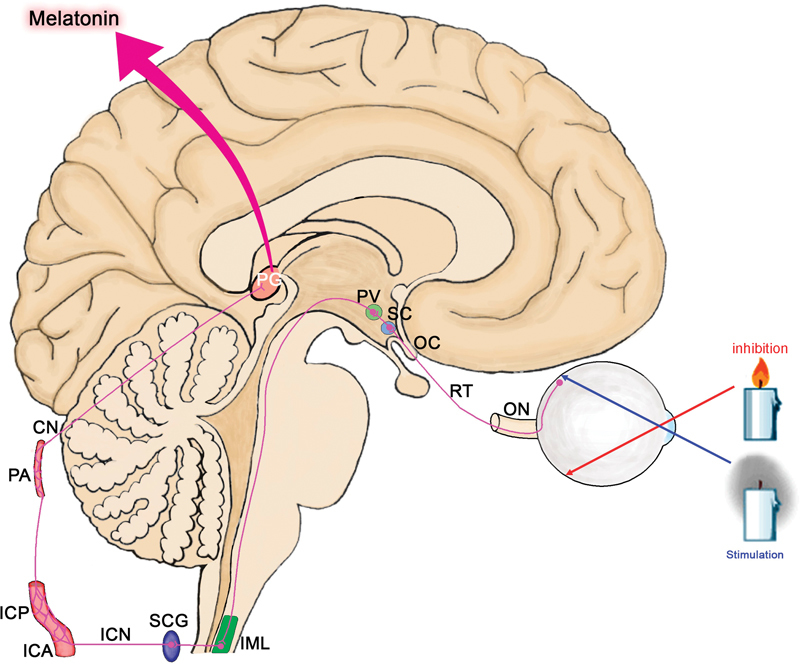
Anatomy of pathway of stimulation of the pineal gland. CN, conarii or pineal nerve; ICA, internal carotid artery; ICN, internal carotid nerve; ICP, internal carotid plexus; IML, intermediolateral column; OC, optic chiasm; ON, optic nerve; PA, pineal artery; PG, pineal gland; PV, paraventricular nucleus; RT, retinohypothalamic tract; SC, suprachiasmatic nucleus; SCG, superior cervical ganglion.

**Fig. 2:**
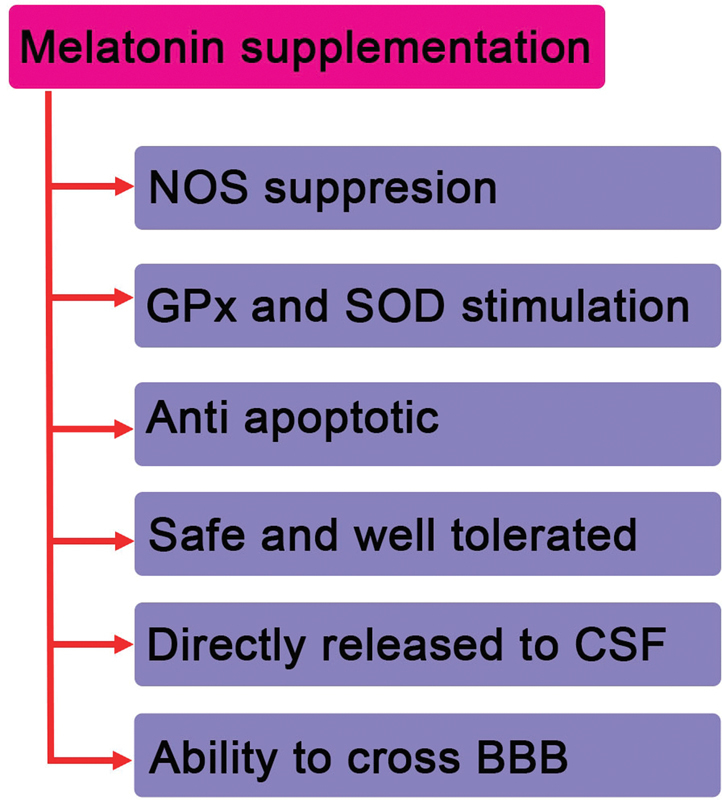
Possible effects of melatonin supplementation. Abbreviations: BBB, blood-brain barrier; CSF, cerebrospinal fluid; GPx, glutathione peroxidase; NOS, nitric oxide synthase; SOD, superoxide dismutase.

The pineal gland or epiphysis cerebri is an endocrine, pine cone–shaped organ located in the center of the brain, posterior to third ventricle, just above superior colliculi ( Fig. 1 ). MLT, a derivative of tryptophan, is produced by the pineal gland in response to incident light. Information about incident light comes from retinal ganglion cells via the retinohypothalamic tract. This tract courses within the optic nerve and optic chiasm and terminates in the suprachiasmatic nucleus of the hypothalamus, which is the circadian pacemaker of the brain, producing day–night impulses and sustaining the “body clock.” Impulses from the suprachiasmatic nucleus are transmitted to the paraventricular nucleus of hypothalamus and end in the preganglionic sympathetic neurons in the intermediolateral column. Postganglionic neurons from the superior cervical ganglion constitute the internal carotid nerve. The internal carotid plexus arising from the internal carotid nerve surrounds the internal carotid artery and passes into the cranial cavity via the carotid canal. This plexus gives subplexuses that carry postsynaptic sympathetic fibers around arteries. A kind of sympathetic plexus around the pineal artery gives nerves to the pineal gland, a pineal or conarii nerve. 6 Noradrenaline is released from these sympathetic nerve endings at night, stimulating postsynaptic adrenergic receptors on the pinealocytes and thus causing synthesis of intracellular cAMP, which activates serotonin N-acetyltransferase, resulting in the rhythmic synthesis and secretion of MLT ( Fig. 2 ). 7 The histology of the pineal gland differs markedly from that of the CNS. It consists of pinealocytes, mostly composed of lobules, with some disseminated in rosette-like structures inside a fibrillary background. The last part contains astrocytes. 8

## Effects of Melatonin in Peripheral Nerve Injury

Despite numerous exploratory studies and clinical observations, there is still no clear treatment strategy for improving peripheral nerve recovery. Also, the role of MLT in nerve degeneration and regeneration is not completely understood. Specific understanding of peripheral nerve science is crucial for comprehending nerve degeneration and recovery. In the clinical setting, the capacity to control nerve biology at the cell level greatly improves the chances of nerve recovery. Various factors such as ischemia and neuroma formation can alter the outcomes of clinical practice.

Nevertheless, some studies have begun to reveal that the pineal neurohormone MLT is neuroprotective and antioxidative. It can reduce oxidative stress by stimulating antioxidative enzymes such as superoxide dismutase (SOD), catalase (Ct), peroxidase, and ascorbate peroxidase. 9 10 11 Endogenous and exogenous MLT is effective as a direct and indirect antioxidant. 12 13 14 15 16

**Fig. 3:**
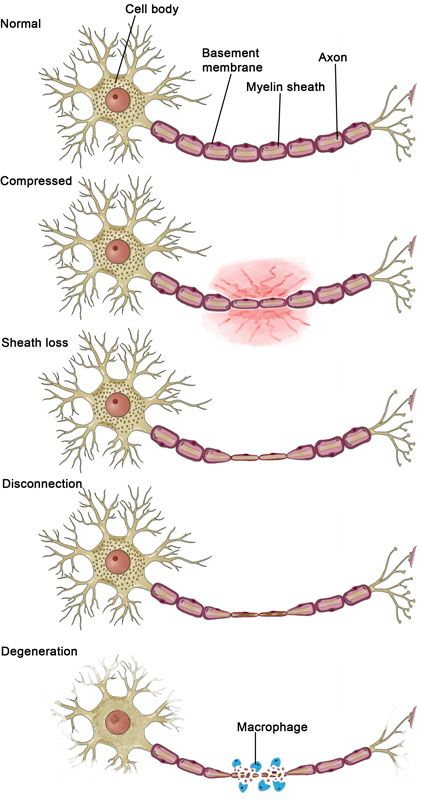
Various types of peripheral nerve injuries including compression, disconnection, and degeneration.

Wallerian degeneration, also known as anterograde or orthograde degeneration, occurs after axonal injury in both the PNS and the CNS. Axonal degeneration is followed by degradation of the myelin sheath ( Fig. 3 ). Activation of Schwann cell proliferation is crucial for axonal guidance and successful nerve regeneration following PNI. 14 Considering the importance of MLT in regulating central glial cell proliferation, Chang et al determined its effect on promoting Schwann cell proliferation and improving nerve regeneration after PNI. Their results indicated that therapeutic use of MLT could be a promising strategy for counteracting PNI-induced neuronal disability. 14

Furthermore, some studies have shown the effect of MLT via surgical removal of the pineal gland, called pinealectomy (Px).

In 2006, a research team of the senior author (Mehmet Turgut) of this article investigated the effects of neonatal Px on peripheral nerve ultrastructure in chickens. 15 16 Their results indicated that neonatal Px caused increases in axon number, thickness of the myelin sheath, and cross-sectional area of the axon. 15 16 Furthermore, their biochemical data concerning hydroxyproline in collagen supported the role of MLT in treating oxidative neuronal damage following ischemia or trauma, and their reports clearly demonstrated that the collagen content of the sciatic nerve was higher in Px chickens than in control animals. 15 16

## Novel Effects of Melatonin on Nervous System Development

One of our coauthors studied the alterations in offspring rat cerebellum induced by maternal exposure to carmustine-[1,3-bis (2-chloroethyl)-1-nitrosourea] (BCNU) and investigated the effects of exogenous MLT on cerebellar BCNU-induced cortical dysplasia, using histological and biochemical analyses. 3 They found that MLT decreased the malondialdehyde (MDA) level in the BCNU group. 3 Erdogan et al and Koch et al also demonstrated that MLT administration protected against the decrease in cannabinoid-1 receptor (CB1R) expression, which is involved in the development of the brain cortex. 17 18 MLT is prophylactic in decreasing the vulnerability of the brain. It is accepted that it protects against harmful effects in epilepsy patients. Uyanikgil et al, Molina-Carballo et al, and Uz et al demonstrated that Px increased, and exogenous MLT decreased, the negative effects of epileptiform activity during pregnancy on postnatal cerebellar tissue in rats. 19 20 21 Its protective effect against ischemia-reperfusion (I/R) injury in neural structures and liver tissue has been demonstrated. 22 23

MLT administration reduced vascular endothelial growth factor (VEGF) and nitric oxide (NO) levels as well as the leakage of rhodamine isothiocyanate, and reduced hypoxia-associated injury, in the developing hippocampus. 24 It has the potential to protect neurons and other elements in the developing hippocampus, as it reduces VEGF concentration, NO production, and vascular permeability. 24 Additionally, the structural modifications found within the dendrites and axons were reversed by MLT treatment. 24

## Free Radical Scavenging Effects of Melatonin in Peripheral Nerve Injury

In some recent studies using the sciatic functional index for nerve recovery, MLT application was shown to be a promising approach for the treatment of peripheral nerve crush injuries. 25 26 It also decreased the collagen content of the wound, as Px caused an elevated collagen content, and MLT application following Px counteracted this. MLT could therefore be expected to minimize the development of scar tissue and posttraumatic neuroma. It also affects the morphological features of nerve tissue and has pain-relieving effects in degenerative diseases of the peripheral nerves. It has a positive effect on axon size and development after peripheral nerve stress: both experimentally and clinically, MLT has been shown to enhance axon length and sprouting after peripheral nerve traumas. Clinically, the positive effect of MLT on neuroma formation and nerve regeneration seems particularly attractive. 15 27 Using an experimental PNI model, Atik et al suggested that the powerful antioxidant and cell-protective effects of MLT could result from mimicking calcium channel blockers. 28 It is therefore believed that boosting peripheral nerve repair with MLT could be a worthwhile option, in addition to other treatment modalities, in cases of MLT deficiency.

Singlet oxygen ( ^1^ Δ _g_ O _2_ ) is an extremely reactive form of oxygen that can be produced by living cells and is a probable cause of cytotoxicity. MLT has been reported to possess potent antioxidant activity and to be capable of scavenging ^1^ Δ _g_ O _2_ . Cagnoli et al investigated whether it can reduce the neurotoxic action of ^1^ Δ _g_ O _2_ . 29 The cytotoxic effect of singlet oxygen was studied in primary cultures of cerebellar granule neurons pretreated with a photosensitive dye, rose bengal, and exposed to light to initiate the generation of ^1^ Δ _g_ O _2_ . 29 They found that this procedure triggered neuronal death, preceded by mitochondrial permeation and apoptosis. 29 Apoptotic DNA fragmentation was determined in situ by a terminal deoxynucleotidyl transferase assay; cell death was assayed using 0.4% trypan blue, and MLT afforded neuroprotection. 29 In a cell-free system, central and peripheral, MLT also protected the enzyme creatine kinase from rose bengal–induced inactivation. 29 The results suggest that MLT can counteract the cytotoxic effect of singlet oxygen. 29

The actions of MLT in free radical scavenging and decreasing NO synthase (NOS) activity can diminish the harmful effects of reactive oxygen. 30 31 32 A possible protective effect of MLT as an antioxidant agent and an inhibitor of neuronal NOS (nNOS) was investigated on spinal motoneurons after axonal injury. 33 It was found in this experiment that nNOS might not be involved in neuronal death or survival. 33 Other authors have reported conflicting results concerning the effects of MLT on nNOS activity. 34 35 36 Apoptotic events after sciatic axotomy and after administration of MLT were investigated experimentally in the spinal cords of neonatal rats. 34 It was suggested that MLT did not alter NOS expression and did not depend on calcium to change NOS activity. 35 Further studies are needed to understand the effect of MLT on nNOS activity in PNI models. Interestingly, a study investigating the neuroprotective effect of various doses of MLT on the lesioned hypoglossal neurons after peripheral axotomy revealed that injury-induced neuronal NADPH-d/NOS expression in the hypoglossal motor neurons can be reduced. It was therefore suggested that MLT is a useful secretory product in reducing oxidative stress after PNIs. 36

Shokouhi et al investigated the neuroprotective effects of MLT on neural fiber injury and lipid peroxidation after blunt sciatic nerve trauma; they found that a low dose reduces trauma-induced myelin breakdown and axonal changes in the sciatic nerve. 37 Stavisky et al found that a significantly higher percentage of crushed rat sciatic axons can be repaired in vitro and/or in vivo by plasmalemma fusion following exposure to MLT. 38

Erol et al observed that both MLT and octreotide gave benefits in an spinal cord injury (SCI) model, and MLT had the more pronounced beneficial effect. 39 Their biochemical and histopathological findings were correlated and significant. 39 For this reason, MLT and octreotide could have the potential to upregulate antioxidant defense systems and be beneficial for patients who sustain SCI. 39 40

I/R produces free radicals leading to lipid peroxidation and nervous tissue injury. The free radical scavenging and antioxidant effects of MLT have been shown to diminish I/R injury in many tissues. 41 The protective effect of MLT was investigated in rats subjected to 2 hours of sciatic nerve ischemia followed by 3 hours of reperfusion. I/R elevated the concentration of MDA significantly, while SOD levels were reduced. MLT treatment reversed the I/R-induced increase in MDA and the decrease in SOD levels. 41 It salvaged the nerve fibers from ischemic degeneration. 41 Histopathological findings in samples from the MLT-treated animals indicated less edema and less injury to myelin sheaths and axons than in the control samples. 41 The results suggest that administration of MLT protects the sciatic nerve from I/R injury, and this could be attributed to its antioxidant property. 41

Data from the literature, together with our studies using a different kind of experimental injury model, showed that MLT has positive effects on the number of axons and the thickness of the myelin sheath by inhibiting collagen accumulation and neuroma formation following traumatic events to peripheral nerves, and on the myelination of a developing peripheral nerve after intrauterine ethanol exposure. 42 As a result, the minimal dose of MLT necessary for an effect on peripheral nerve regeneration can be ascertained. Ulugol et al determined the effects of intracerebroventricular (i.c.v.) and intraperitoneal (i.p.) MLT on mechanical allodynia and thermal hyperalgesia in mice with partial tight ligation of the sciatic nerve, and the influence of the NO precursor L -arginine and the opiate antagonist naloxone on those effects. 43 A plantar analgesic meter was used to assess thermal hyperalgesia, and nerve injury–induced mechanical hyperalgesia was assessed with von Frey filaments. 43 One to 5 weeks after the surgery, marked mechanical allodynia and thermal hyperalgesia developed in the neuropathic mice, but MLT at higher doses blocked the thermal hyperalgesia but not the mechanical allodynia. 43 Administration of both L -arginine and naloxone, at doses that produced no effect on their own, partially reversed the anti-hyperalgesic effect of MLT. 43 These results suggest that although MLT has different effects on neuropathic pain-related behaviors, the L -arginine–NO pathway and opioidergic system are involved in its anti-hyperalgesic effect in nerve-injured mice. 43

## Current Studies

Recently, the protective effects of MLT have been supported from many perspectives. The interaction between the vagus nerve and MLT was studied and the protective effect of MLT was demonstrated. 44 These researchers compared the effects of aminoguanidine, MLT, and methylprednisolone; MLT supported regeneration after peripheral facial nerve neurorrhaphy. 45 Administrating MLT after stripping of the epineural vessels in a model of sciatic nerve injury supported axonal regeneration, reduced oxidative stress, and improved functional recovery. 46 More recently, Moretti et al demonstrated for the first time that MLT reduces injury to the blood–brain barrier after an excitotoxic insult and their results were supported by gene analyses. 47

## Conclusion

It has been shown that MLT affects the nervous system via free radical scavenging and antioxidant properties. Notwithstanding these studies, there is no obvious procedure for using MLT in clinical applications. Nevertheless, we believe that research on MLT can standardize its use. Almost every month new research is being published and giving a new impulse to the field, but further experiments and randomized controlled clinical studies are needed to standardize the clinical use of MLT. The future search for novel drug treatment for PNI is critical. Despite some use of MLT pills, deployment of MLT as a neuroprotective agent is still not close to clinical practice, but should become possible in future, as safe and effective procedures are discovered and developed.
